# Warm and dry weather accelerates and elongates *Cladosporium* spore seasons in Poland

**DOI:** 10.1007/s10453-016-9425-7

**Published:** 2016-02-12

**Authors:** Idalia Kasprzyk, Boguslaw Michal Kaszewski, Elzbieta Weryszko-Chmielewska, Malgorzata Nowak, Aneta Sulborska, Joanna Kaczmarek, Agata Szymanska, Weronika Haratym, Malgorzata Jedryczka

**Affiliations:** Department of Environmental Biology, University of Rzeszow, Zelwerowicza 4, 35-601 Rzeszow, Poland; Department of Meteorology and Climatology, Maria Curie-Sklodowska University, Krasnicka 2cd, 20-718 Lublin, Poland; Department of Botany, Lublin University of Life Sciences, Akademicka 13, 20-950 Lublin, Poland; Laboratory of Aeropalynology, Faculty of Biology, Adam Mickiewicz University, Poznan, Poland; Institute of Plant Genetics, Polish Academy of Sciences, Strzeszynska 34, 60-479 Poznan, Poland

**Keywords:** Climate change, Environment, Hirst-type spore sampler, Temperature rise, Weather conditions

## Abstract

Temperature is the environmental factor that systematically changes for decades and, as in plants and animals, can significantly affect the growth and development of fungi, including the abundance of their sporulation. During the time of study (2010–2012), a rapid increase in air temperature was observed in Poland, which coincided with the substantial decrease in rainfall. The increase in annual mean temperatures at three monitoring sites of this study was 0.9 °C in Lublin and Rzeszow (east Poland) and 2.0 °C in Poznan (west Poland). Such warming of air masses was comparable to the average global air temperature rise in the period of 1880–2012 accounting for 0.85 °C, as reported by the Intergovernmental Panel on Climate Change. Moreover, there was a substantial decrease in rainfall, ranging from 32.7 % (Poznan) to 43.0 % (Rzeszow). We have demonstrated that under such conditions the mean and median values of total *Cladosporium* spore counts significantly increased and the spore seasons were greatly accelerated. Moreover, earlier start and later end of the season caused its extension, lasting from over 20 days in Rzeszow to around 60 days in Lublin and Poznan, when the cumulative amount of 5–95 % of spores was considered. The time of reaching the cumulative amount of 50 % of spores was up to 25 days earlier (difference in Poznan between 2010 and 2012). There was also a striking acceleration of the date of the maximal *Cladosporium* spore concentration per cubic metre of air (26 days for Lublin, 43 for Poznan and 56 for Rzeszow).

## Introduction

The ascomycetous fungi of the genus *Cladosporium* (Davidiellaceae, Capnodiales, Dothideomycetes) occur worldwide in a wide range of habitats as plant pathogens, secondary parasites and necrotrophs (O’Donnel and Dickinson 1980; Collemare et al. 2014). They colonize decaying plant material, mainly leaves and stems, but have also been isolated from other materials such as soil, stones, concrete, bricks as well as textiles, paper and leather (Gutarowska 2014). The fungi tolerate a wide range of environmental conditions. The optimal temperature of their development is 18–28 °C, but they still slowly grow around 0 °C (Gravesen 1979). Laboratory experiments performed by Damialis et al. (2015a) showed positive correlation between temperature and the growth of mycelium as well as the spore production.

*Cladosporium* belongs to ‘dry-spore’ group of taxa; almost all studies identify temperature as the most important factor influencing its growth, sporulation and concentration in the air (Pady et al. 1969; Rodriguez-Rajo et al. 2005; Stepalska and Wolek 2005; Sánchez Reyes et al. 2009; Grinn-Gofron and Bosiacka 2015). In contrast, Papkour et al. (2015) found that precipitation had a stronger effect than temperature on the concentration of *Cladosporium* spores in the air. Positive correlation of rainfall with the spore count of *Cladosporium* was also reported by Recio et al. (2012). During and after rainstorms, increasing air humidity resulted in low levels of spore counts in the air (Grinn-Gofron and Strzelczak 2013) even though higher temperatures and ozone levels before storms caused an increase in *Cladosporium* spore concentration. In common with most airspora, wind speed and direction also play an important role in the distribution of *Cladosporium*, and the source of spores is detectable using aerobiological methods (Recio et al. 2012; Grinn-Gofron et al. 2015; Sadyś et al. 2015).

Statistical models have been designed for calculating and accurately predicting the patterns of spore release in many (Grinn-Gofron et al. 2011; Kasprzyk et al. 2015), but not all fungal taxa (Havis et al. 2015; Jedryczka et al. 2015). Moreover, the abundance of airborne particles sometimes depends on weather of the actual day, as well as the previous periods. For *Cladosporium*, Grinn-Gofron et al. (2011) demonstrated the impact of meteorological parameters observed over three preceding days. In all cases studied by these authors, temperature and dew point caused an increase in *Cladosporium* spore number in the air, whereas a decrease in spore concentration was related to decreasing humidity as well as both average and maximum wind speed. The most important variable of the forecasting model developed for Szczecin, Poland, was the dew point temperature of the previous day. Antecedent weather parameters were also significant for the concentration of *Cladosporium* spores in New York and Toronto (Papkour et al. 2015). The total precipitation during 2 months prior to sampling, done in September, was negatively correlated to observed fungal spore concentrations at both locations. Considering an increase in number of drought events in summer, the authors forecasted an increase in airborne fungal concentrations in future years.

The concentration of biological particles in the air depends on a complex of meteorological parameters (Kasprzyk et al. 2013). In Stockholm, Sweden, the optimum condition for *Cladosporium* sporulation was a combination of temperatures above 15 °C and precipitation (Hjelmroos 1993). An interesting approach to this problem was proposed by Rodriguez-Rajo et al. (2005), who created bioclimatic markers or indices, called continentality, thermicity and ombrothermic indices. An increase in the continentality index was related to the increase in *C.cladosporioides* spore concentration.

Regional forecasts of temperature changes show significant and constant warming of the entire globe, including Poland (Christensen et al. 2007). The forecasts for warming in Europe concern all seasons, but the strongest rise in temperature is forecasted for winter. Forecasts for global temperature increase, including Poland, show a 2–4 °C rise in air temperature by the end of twenty-first century. According to the IPCC (2013), the average global air temperature for the period 1880–2012 has risen by 0.85 °C, at the rate of 0.13 °C per 10 years. In the case of precipitation, the trends were not so clear. Poland is under the influence of polar maritime air mass, from the north, for more than 60 % of the year. Polar continental air is noted on 9–13 % of days, and subtropical air, on 1–4 % of days per year. There are major regional differences in the occurrence of air masses over the country. In west Poland, the polar maritime air mass accounts for more than 70 % of days per year, but in the east and north-east, it drops down to 63–64 %. Similar differences, but with the opposite geographical gradient, are observed for the polar continental air mass. The average annual air temperature in Poland (apart from mountainous areas) ranges from approximately 6.2 °C in the north-east to 8.7 °C in north-west of the country (Wos 2010). During spring and summer, the lowest air temperature values are observed in northern Poland, while the highest are recorded in the central parts. In the spring and summer months, the isotherms are nearly latitudinal (E–W), and in the autumn and winter, they are meridional (S–N). In Poland, over the period of 1951–2010, a statistically significant increase in mean annual air temperature was observed and the average rate of warming slightly exceeded 0.2 °C per decade. An upward trend was significant in the spring (0.36 °C decade^−1^), especially in February (more than 0.5 °C/10 years), and in summer (almost 0.2 °C/10 years), both in July and in August (Wojcik and Mietus 2014).

Precipitation in Poland is highly variable in time and space. The distribution of rainfall is largely affected by altitude, the shape of the terrain and the exposure of slopes in relation to the prevailing direction of air masses. In most areas of Poland, the precipitation ranges from 500 to 700 mm. The highest rainfall is noted in Pomeranian Lakeland, and the lowest, in Great Poland (Poznan) and Mazovia (Warsaw). The annual rainfall over the period 1961–2008 varied significantly in terms of both value and direction of change. Rainfall increases were recorded in Pomerania, the Baltic Sea coast (Maritime region) and the south-eastern parts of Poland (Rzeszow). The central and southern parts of the country had a systematic decrease in precipitation, especially in Lower Silesia and the Sudethian Foothills (Marosz et al. 2011).

Climate change has a huge effect on the biosphere, but it is difficult to predict the exact change of the structure of current ecosystems and the behaviour of living organisms. Studies on the response of plants to a significant rise in temperature are intensively conducted (Goldblum and Rigg 2005; Chmielewski et al. 2005; Bortenschlager and Bortenschlager 2008). Some attention is given to fungi (West et al. 2012), but the investigations of climate change impact on airborne spores are scarce (Damialis et al. 2015a; Kaczmarek et al. 2015). Meanwhile, temperature rise changes the phenology of many living organisms, including fungi. Some studies have indicated an earlier start of fruiting of macrofungi in Norway and the UK. Kauserud et al. (2010), who studied spring-fruiting fungi, forecasted over 3 days of acceleration of their maturation per decade during 1960–2007. The reaction of airborne spores to climate change is difficult to anticipate, as their concentration in air is strongly influenced by a complex of meteorological parameters. The monitoring of airborne spores conducted for 15 years in Thessaloniki, Greece, showed decreased concentration of many fungal taxa (Damialis et al. 2015b). Seasons tended to start later and last shorter. However, this trend was not found for *Cladosporium*; on the contrary, the occurrence of spores started and ended earlier and the day of peak spore concentration was delayed. Experiments imitating the increasing temperatures showed intensive mycelial growth in several species such as *Alternaria alternata*, *Botrytis cinerea*, *Aspergillus niger* as well as *Cladosporium oxysporum* and *C. cladosporioides* (Damialis et al. 2015a). Furthermore, apart from *C. cladosporioides*, spore production decreased in these species.

Although members of the genus *Cladosporium* are the most widespread and common causes of aerially dispersed allergy (Knutsen et al. 2012), the effects of warming on the sporulation and spore abundance of these fungi remain unknown and require further studies. This paper presents the outcome of investigations on the effect of raised temperatures and decreased precipitation on spore production and the patterns of *Cladosporium* spore seasons conducted by three separate research teams in cities located in different weather zones of Poland. According to our hypothesis, warming has a great impact on *Cladosporium* spore seasons and most probably results in the increase in spore numbers and their higher concentration.

## Materials and methods

### General description of weather at monitoring sites

**Fig. 1:**
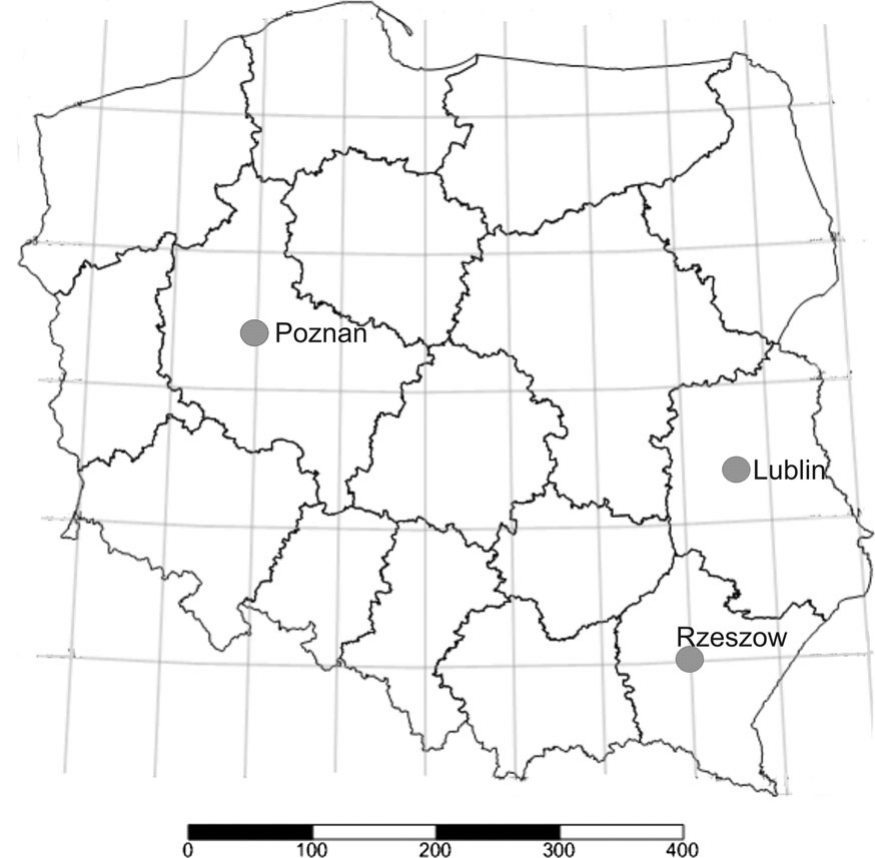
Location of experimental sites in Poland: 1) Poznan, 52°28′02″N; 16°55′27″E, 2) Lublin, 51°14′37″N; 22°32′25″E, 3) Rzeszow, 50°02′24″N; 22°00′00″E

The experiment was located both in west of Poland (Poznan), which resembles the intermediate–mild weather encountered in the west of Europe, and in the east (Lublin and Rzeszow), with more harsh and continental weather, similar to that of Eastern Europe (Fig. 1).

#### Lublin

According to average temperatures based on 60-year temperature records (1951–2010), the warmest month was July (19.0 °C) and the coldest, January (−2.8 °C). Substantial differences in temperature between summer and winter indicate the continental climate. Average temperatures were lower when compared to Poznan and Rzeszow. The average annual rainfall was 551 mm. Maximum precipitation was recorded in July (79 mm), while minimum rainfall, in January (28 mm) (Piotrowska and Kaszewski 2011). According to Dmochowska (2012), in 2012 the agricultural land constituted 63.2 % of the area of Lublin region.

#### Poznan

The lowest monthly average air temperatures in the last 60 years (1951–2010) were encountered in January (−1.0 °C), and the highest temperatures were in July (18.2 °C). The weather was under the influence of the oceanic climate characterized by frequent precipitation of low intensity. The average annual amount of precipitation was 634 mm, with a maximum rainfall in July (76 mm). In the area of the city, westerly winds blew most frequently at a speed of 2–10 m s^−1^. Poznan had the highest number of days (64) and time (280 h) with mist (Wos 2010). Poznan is the capital of Wielkopolska region (Great Poland), located on North European Plain. In 2012, the land used to grow agricultural crops was 60.1 % of the total area of this region (Dmochowska 2012).

#### Rzeszow

The average annual temperature in the last 60-year period (1951–2010) was 8.9 °C. The highest average annual temperatures were observed in July (19.6 °C), and the lowest, in January (−2.1 °C). Prevailing winds blew from west and north-west. East winds accounted for about 13 % of the total and primarily blew in winter. Rzeszow is the capital of Podkarpacie region (Carpathian Foothills). The area is covered by many forests, and the agricultural land is the lowest among the studied regions—agricultural areas constitute only 52.5 % of the region (Dmochowska 2012).

### Weather during the time of study

The investigations were conducted over three consecutive years (2010–2012) from 1 April to 30 September. In all cities, relationships between the concentration of spores and meteorological factors were analysed using the following weather data: mean, maximum and minimum temperature (°C), relatively air humidity (%), rainfall (mm), and wind speed (m s^−1^). In Lublin, the meteorological data were obtained from the Meteorological Observatory of the Meteorology and Climatology Department, the Maria Curie-Sklodowska University. The meteorological station was located at a distance of 1.5 km from the spore sampling site. In Rzeszow, meteorological data were obtained from the Rzeszow–Jasionka Airport located 10 km from the city centre. Meteorological data from Poznan were obtained from the Lawica Airport, situated 8.5 km south-west of the sampler, belonging to the Institute of Meteorology and Water Management. It was obtained using the Vaisala Automatic Weather Station MAWS 301 (Vaisala Group, Helsinki, Finland). The mean yearly weather parameters at the monitoring sites are presented in Table 1.

**Table 1:** Mean monthly weather parameters in 2010, annual parameters in 2010–2012 and differences in parameter values at monitoring sites during the study

Parameter	Lublin				Poznan				Rzeszow			
	*T*_mean_ (°C)	*T*_max_ (°C)	*T*_min_ (°C)	Rain (mm)	*T*_mean_ (°C)	*T*_max_ (°C)	*T*_min_ (°C)	Rain (mm)	*T*_mean_ (°C)	*T*_max_ (°C)	*T*_min_ (°C)	Rain (mm)
*April*												
2010	9.86	5.71	14.45	22.4	9.08	3.17	14.59	43.4	9.16	2.91	14.92	50.8
2011–2010^a^	1.14	0.67	1.14	9	2.96	2.58	3.21	−32.7	1.07	0.74	1.80	−6.9
2012–2010^b^	0.48	0.27	0.39	4.7	0.56	0.56	0.30	−20.5	1.54	0.46	−0.53	−24.91
2012–2011^c^	−0.66	−0.40	−0.75	−4.3	−2.39	−2.01	−2.92	12.2	0.47	−0.28	−2.33	−18.01
*May*												
2010	14.88	11.74	18.61	139.2	11.76	7.39	15.63	110.7	14.19	9.96	18.84	172.72
2011–2010	0.22	−1.87	1.09	−106.2	3.14	0.06	5.44	−99.8	−0.19	−3.77	2.13	−110.24
2012–2010	1.29	−0.53	2.19	−106	4.13	2.46	6.06	−50.8	0.58	−1.53	1.50	−116.85
2012–2011	1.07	1.34	1.10	0.2	0.99	2.41	0.62	49	0.77	2.23	−0.63	−6.61
*June*												
2010	18.71	14.32	22.53	58.7	17.73	10.73	23.50	17	17.94	12.10	23.05	136.91
2011–2010	0.51	0.45	1.12	13.4	1.51	1.98	1.75	34.6	0.34	0.13	1.10	−47.76
2012–2010	−0.63	−0.20	−0.50	2.3	−1.09	1.21	−1.81	95.3	0.26	0.03	1.04	−59.94
2012–2011	−1.15	−0.65	−1.61	−11.1	−2.59	−0.76	−3.56	60.7	−0.08	−0.11	−0.07	−12.18
*July*												
2010	22.13	17.60	26.47	51.1	22.41	15.78	28.09	80.5	20.62	14.73	26.80	200.39
2011–2010	−3.35	−2.21	−3.51	81.3	−4.19	−2.35	−5.53	102.9	−2.24	−0.81	−3.19	27.45
2012–2010	0.04	−0.25	0.35	−8.9	−2.59	−0.77	−3.43	71.6	0.28	1.15	0.79	−146.81
2012–2011	3.39	1.96	3.85	−90.2	1.60	1.58	2.10	−31.3	2.52	1.96	3.98	−174.26
*August*												
2010	20.42	16.50	25.21	131.3	18.94	14.30	23.40	154.4	19.63	13.77	25.56	74.94
2011–2010	−1.07	−1.43	−1.23	−90.8	0.29	−0.37	0.79	−105.9	−0.36	−0.59	−0.24	−38.87
2012–2010	−0.66	−1.11	−0.74	−91.2	0.25	−0.72	1.44	−115.5	−0.70	−0.95	0.14	−18.54
2012–2011	0.41	0.32	0.49	−0.4	−0.04	−0.35	0.65	−9.6	−0.34	−0.36	0.38	20.33
*September*												
2010	12.39	9.70	16.25	105	12.83	8.32	17.23	74.2	12.69	7.91	17.37	112.52
2011–2010	3.32	1.61	4.38	−101.8	2.83	1.60	4.04	−49.1	3.03	0.80	4.98	−103.88
2012–2010	2.98	1.75	4.09	−69.9	2.05	1.39	2.77	−41.7	2.09	−0.05	2.48	−73.39
2012–2011	−0.34	0.14	−0.29	31.9	−0.78	−0.21	−1.27	7.4	−0.95	−0.85	−2.50	30.49
*Year*												
2010	7.5	11.7	3.6	761	8.0	12.1	3.4	723	8.2	12.7	3.5	981
2011	8.4	12.9	4.2	505	10.0	14.4	5.1	484	9.1	13.7	3.9	616
2012	8.2	12.6	3.9	512	9.4	13.7	5.0	678	8.9	13.7	3.8	559

Positive number—value of weather parameter is higher in the following year

Negative number—value of weather parameter is lower in the following year

^a^Difference of the value of weather parameter between 2011 and 2010

^b^Difference of the value of weather parameter between 2012 and 2010

^c^Difference of the value of weather parameter between 2012 and 2011

### Weather change during the time of study

The variable rate of inflow of air masses in the period of study and their time of exposure over a given area caused a markedly different distribution of air temperature and precipitation, between cities and years/months (Table 1).

In 2010, the distribution of mean annual air temperature in Poland (except for mountain areas) showed an increase of 6–7 °C at the Mazury Lake District and the eastern part of the Pomeranian Lake District as compared to the years 2011 and 2012. This increase reached over 8 °C in the Sandomierz Basin, the central valley of Vistula and Odra valley. Out of the three stations analysed (Table 1), the highest mean annual air temperature was recorded in Rzeszow (8.2 °C), and the lowest, in Lublin (7.5 °C). In 2011, the temperature range observed over the area of Poland was higher than in 2010. The coldest area was Podlasie region, located in north-eastern Poland (7–8 °C). The temperatures increased towards the west, and in the area of Zielona Gora (central-west), the average increase in the temperature exceeded 10 °C. In 2011, the average annual temperature was the highest in Poznan (10.0 °C), and the lowest, in Lublin (8.4 °C). The temperature distribution in 2012 was similar to that in 2011, with slightly lower values in the north-east and the reduction of the area of western part of Poland with temperatures above 9 °C. Similarly to 2011, the highest average temperature was recorded in Poznan (9.4 °C) and the lowest, in Lublin (8.2 °C).

Distribution of precipitation in Poland in 2010–2012 was very diverse (Table 1). In 2010, the rainfall in Poland was higher than the average perennial. Apart from the mountains, the highest rainfall (900–1100 mm) occurred in the eastern part of the Pomerania and the areas adjacent to the north side of the Carpathians and the Sudethian Foothills. The lowest total rainfall was recorded in the eastern part of Lublin as well as in Great Poland and the northern part of the Mazury Lake District. The highest annual total precipitation occurred in Rzeszow (981 mm) and the lowest, in Poznan (723 mm). The precipitation on almost entire area of the country was significantly lower in 2011 than annual totals. This phenomenon was most evident in Great Poland (Poznan), where the annual rainfall was 484 mm. Similarly to 2010, the highest annual sum of rainfall was observed in Rzeszow (616 mm). In 2012, the lowest rainfall was noted in the southern and eastern part of Great Poland, western Mazovia, and Lublin Upland. The largest rainfall was recorded in the middle of the Pomeranian Lake District. There was a dramatic decrease in rainfall between the southern and northern part of Great Poland and the central part of the Pomeranian Lake District. In contrast to the previous years, the largest annual rainfall was recorded in Poznan (678 mm) and the smallest was in Lublin (512 mm).

### Spore sampling and spore counts

Studies were conducted in accordance with the recommendations of the International Association for Aerobiology (Mandrioli et al. 1998). The volumetric method was applied at all sampling sites, using Hirst-type samplers (Lanzoni VPPS 2000). In Lublin (51°14′37″N; 22°32′25″E) and Poznan (52°28′02″N; 16°55′27″E), the air sampler was located at 18 m above ground level (a.g.l.), while in Rzeszow (50°02′24″N; 22°00′00″E), the trap was placed at a height of 12 m a.g.l. Spores were counted using a light microscope (×400), along one 48-mm-long horizontal line. The resulting number of spores was multiplied by a correction factor and expressed as the number of spores in 1 m^3^ of air per day (s m^−3^).

Data collected included the cumulative number of *Cladosporium* spores per month, the total spore count during the study period as well as the highest daily concentration and the date of its occurrence. Seasons of *Cladosporium* spore occurrence in the air were divided into nine phases. Each phase started when the cumulative percentage from the seasonal total sums of spore concentration reached the appropriate values: 1; 2.5; 5, 25; 50, 75; 95; 97.5; and 99 %.

### Statistical methods

Data were checked for normality using the Shapiro–Wilk test. As the distributions were not normal, nonparametric tests were applied. The synchronization of the patterns of airborne spore occurrence was expressed by the coefficient of Spearman’s rank correlation. This test was also used to evaluate the strength and the direction of the relationships between the spore concentration and meteorological parameters. The Kruskal–Wallis test was applied for comparing the spore concentrations between the cities and the years, and the Dunn’s post hoc test was used for detailed comparisons between pairs of site-years. The statistical hypotheses were tested at *p* ≤ 0.05. In order to distinguish the groups of the cities with the highest similarity with respect to the descriptive statistics, hierarchical clustering analysis was used. Statistical and multidimensional analyses were performed using STATISTICA version 9 (Stanisz 2006).

## Results

**Fig. 2:**
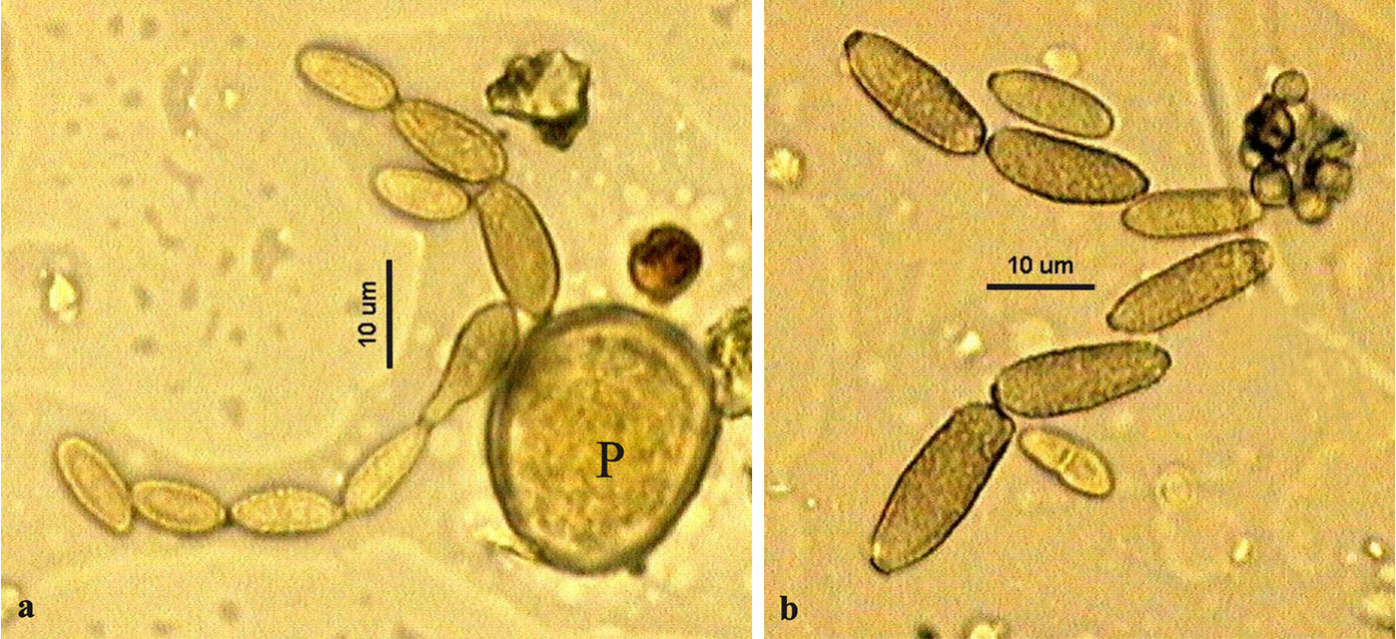
Conidiospores of *Cladosporium* spp. **a** chain of conidiospores on the *left*, the pollen grain of Poaceae (P) on the *right*; **b** a close-up photograph of conidiospores

The conidiospores observed on microscope slides were usually ovoid, ellipsoid, limoniform, subcylindrical to cylindrical-oblong and sometimes also pyriform (Fig. 2a). They often had a distinct ornamentation of the cell wall (Fig. 2b). The sizes varied within the range 4–25 × 3–7 µm. The slides also contained numerous other spores and pollen grains, e.g. of Poaceae family (Fig. 2a). However, the spores of *Cladosporium* outnumbered all other types of spores and pollen grains.

### Characteristics of spore seasons

**Fig. 3:**
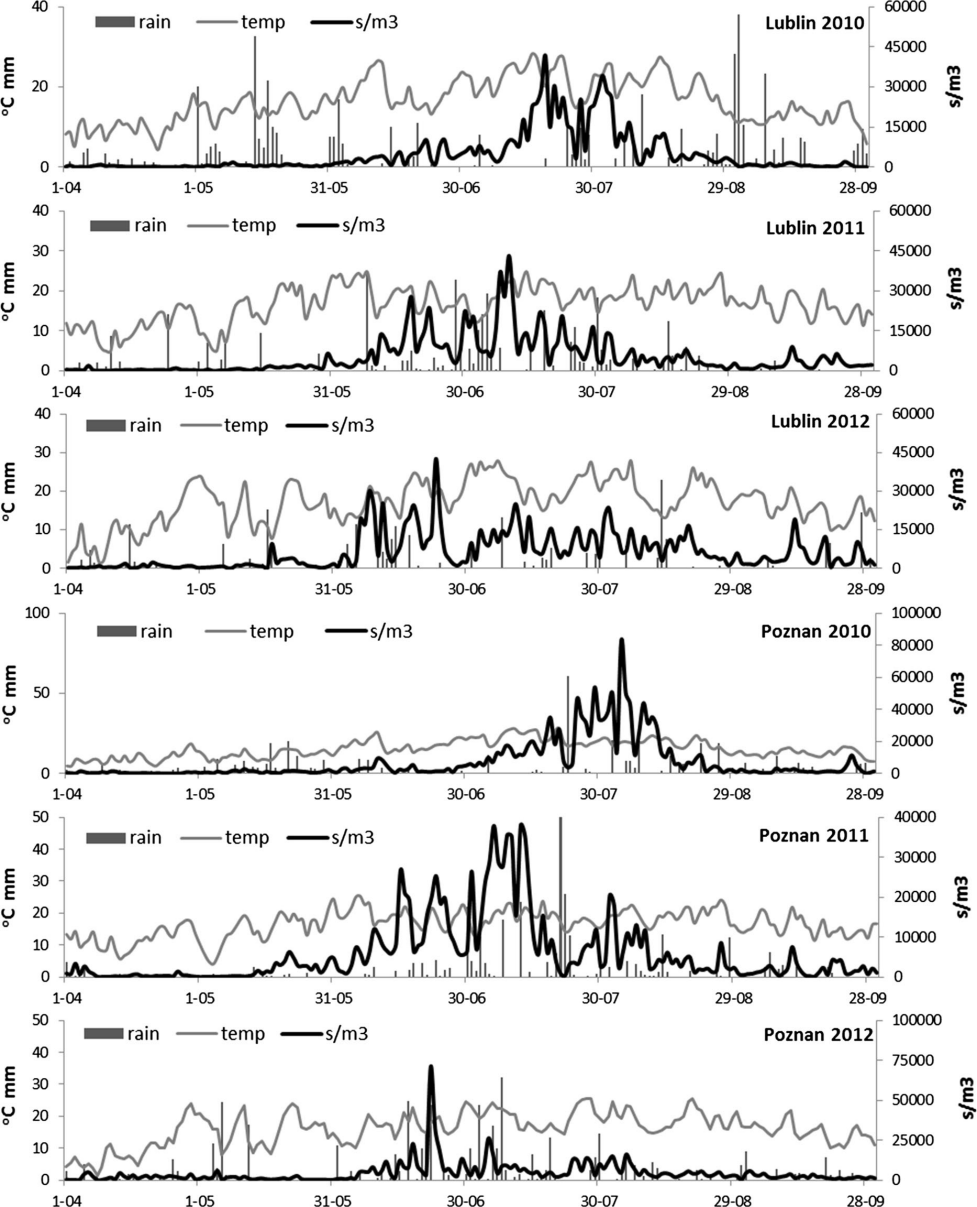

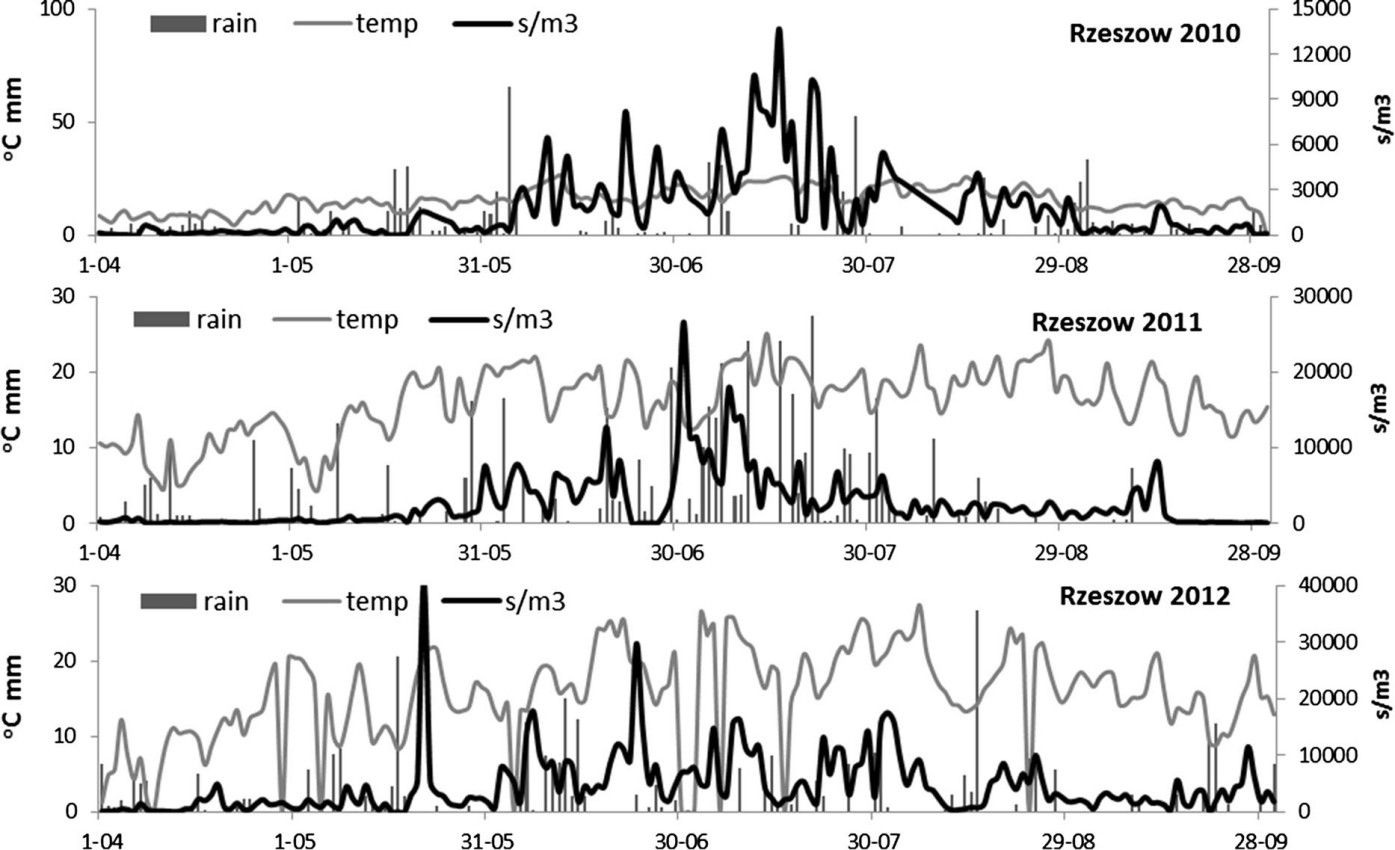
Distribution patterns of the concentrations of conidiospores of *Cladosporium* spp. in air (s m^−3^ *black line*), rainfall (mm, *black bars*) and temperature (°C, *grey line*) in three cities of Poland (Lublin, Poznan and Rzeszow) in 2010–2012

The patterns of *Cladosporium* spore concentrations varied considerably between the monitoring sites, years and parts of the season (Fig. 3). The curves were almost exclusively multimodal, i.e. they had many peaks (in Lublin and Rzeszow in all years and in Poznan in 2010 and 2011). Only the spore release observed in Poznan 2012 concentrated in a definite period of time, with one main peak.

In each city, the periods of the highest *Cladosporium* spore concentrations as well as the daily spore peak in subsequent years were reported earlier than in the previous year (Fig. 3). In 2010 in Rzeszow and Lublin, the maximum concentration of *Cladosporium* spores was noted in July, on the 16 and 19, respectively (Table 2). In Poznan, the same phase was obtained on August 4. In 2011, the highest concentrations of the spores of this fungus in three cities were recorded between July 1 and 10. In Lublin and Poznan, the highest concentrations of the conidiospores of *Cladosporium* were recorded in the last 10 days of June 2012, and in Rzeszow, this phase was reached exactly 1 month earlier, i.e. on May 21.

**Table 2:** Selected descriptive characteristics of the *Cladosporium* spore seasons in three cities of Poland in the period 2010–2012

Monitoring site	Year/parameter	Maximum concentration		Seasonal total s/m^3^
		No. of spores per cubic metre	Date	
Lublin	2010	41632	19 July	860308
	2011	43189	10 July	944181
	2012	42600	23 June	1165517
	Mean	42474	06 July	990002
	Variation coefficient	1.8 %	35 %	15.9 %
Poznan	2010	83102	4 August	1330015
	2011	37892	6 July	1078584
	2012	70954	22 June	835802
	Mean	63983	9 July	1081467
	Variation coefficient	36.6 %	63 %	22.9 %
Rzeszow	2010	13670	16 July	325633
	2011	26636	1 July	484437
	2012	40708	21 May	829028
	Mean	27005	21 June	546366
	Variation coefficient	50.1 %	54 %	47.1 %

**Fig. 4:**
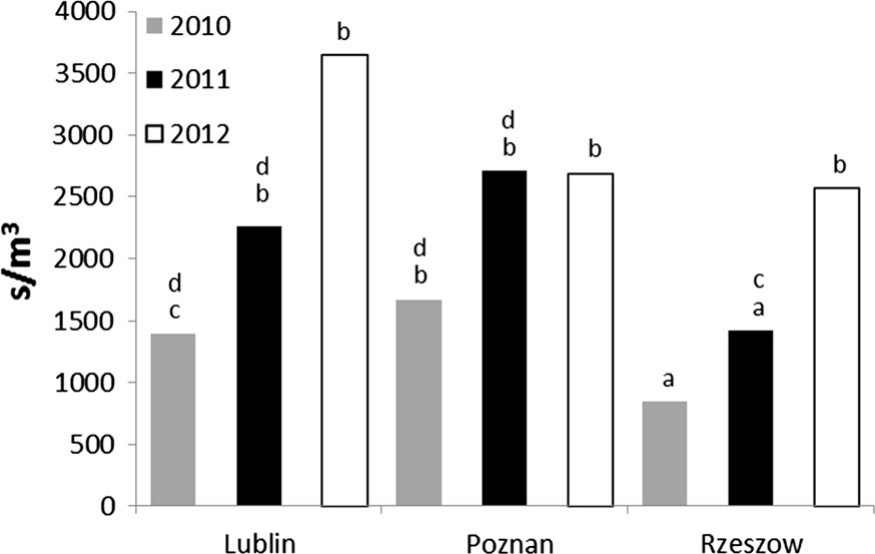
Median values of total spore counts per site-year in 3 years of study (*letters* indicate the homogeneous groups)

**Fig. 5:**
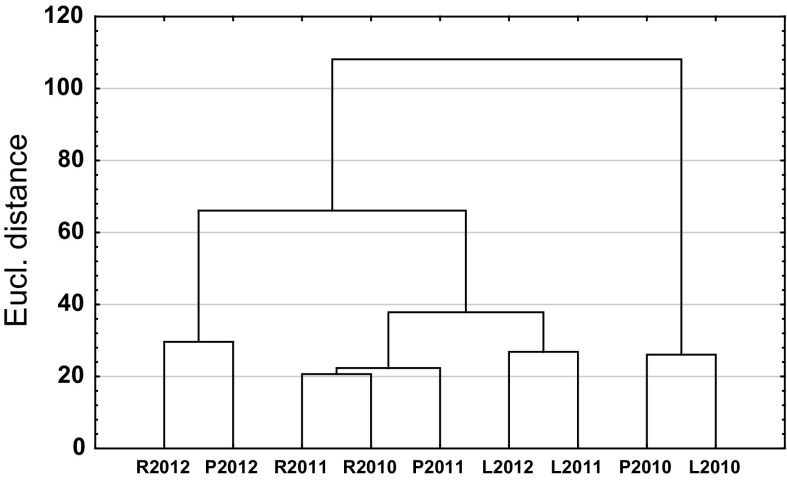
Dendrogram clustering site-years with the highest similarity of the patterns of *Cladosporium* spore seasons (R—Rzeszow, P—Poznan and L—Lublin)

Within 3 years, the daily average concentrations at three sites differed significantly (Kruskal–Wallis *H* = 118.92, *p* = 0.000) (Fig. 4). Median values of total spore concentrations per site-year in 3 years of study increased in each subsequent year except Poznan in 2012. Multiple comparison tests indicated significant differences between both monitoring sites and years as well as between groups of site-years. According to average values, the most homogeneous results originated from Rzeszow in 2010 and 2011 (group ‘a’). These results differed significantly from all others with the exception of Lublin 2010. The second group (‘b’) was composed of the following site-years: Rzeszow 2012, Poznan from all years, and Lublin from years 2011 and 2012. The analysis indicated a similarity between Lublin and Rzeszow in 2011 (Fig. 5).

### Yearly spore concentrations

There were statistical differences in the annual sums of daily concentrations of *Cladosporium* spores at monitoring sites. The 3-year means for Lublin and Poznan were almost twice high as for Rzeszow (Table 2). However, the data obtained in particular years of the study in Lublin and Poznan also significantly differed from each other. In 2010 and 2011, the highest annual sums of spores were recorded in Poznan, and in 2012, this parameter was the highest in Lublin. The development of *Cladosporium* spores in Lublin and Rzeszow, which are located at the closest distance (170 km), was most favourable in 2012, whereas in Poznan the most conducive year for the formation of spores of this fungus was 2010.

### Maximal spore concentrations

The highest concentrations of *Cladosporium* spores were observed in June and July (Table 2). There were clear differences in this parameter between the particular years, with the exception of 2012, when similar values were noted in Lublin and Rzeszow. The highest concentrations of spores were recorded in Poznan, and the lowest, in Rzeszow. The maximal concentrations of spores in Lublin slightly exceeded 40,000 s m^−3^ each year; in the other cities, these values were not as uniform. The average values of this parameter in Poznan exceeded by 50 % the maximum concentrations reported in Lublin. Maximal concentrations of *Cladosporium* spores in Rzeszow accounted for about 64 % of the average peak concentrations noted in Lublin.

### Effect of meteorological parameters on *Cladosporium* spore concentrations

Data analysis revealed temperature was the most important meteorological parameter positively affecting *Cladosporium* spore concentrations in the air (Table 3). In all cities, the highest values of correlation coefficients were noted in 2010 and they ranged from 0.844 (*T*_mean_) in Poznan to 0.682 (*T*_min_) in Rzeszow. In the succeeding years, the relationships between temperature and airborne spore concentrations decreased, and in one case (Rzeszow 2012), they were not significant. Mean temperature most strongly affected the number of spores in the air, while maximum temperature had the smallest effect among three temperature parameters. The role of rainfall was unclear; the correlation coefficients were either low or not statistically significant. In Poznan, relationships with rainfall were positive but in Rzeszow in 2010 they were negative. To a small extent, *Cladosporium* spore concentrations were affected by air humidity. These relationships were positive in Lublin and Poznan in 2011, whereas the spore concentrations decreased in high air humidity at all locations in 2012. It was found that the wind speed had a very low effect. The negative correlation coefficients were significant in Poznan 2010 and 2012, but their values were low (Table 3).

**Table 3:** Spearman’s rank correlation coefficients between meteorological parameters and *Cladosporium* spores concentrations (s m^−3^)

	*T*_mean_ (°C)	*T*_min_ (°C)	*T*_max_ (°C)	*H* %	Rain (mm)	Wind speed
Lublin 2010	**0.820**	**0.813**	**0.773**	−0.165	NS	NS
Lublin 2011	**0.597**	**0.670**	**0.531**	**0.436**	NS	NS
Lublin 2012	**0.577**	**0.603**	**0.537**	NS	NS	NS
Poznań 2010	**0.844**	**0.827**	**0.792**	−0.256	NS	−0.208
Poznań 2011	**0.648**	**0.669**	**0.528**	**0.169**	**0.317**	NS
Poznań 2012	**0.559**	**0.590**	**0.506**	NS	**0.164**	−0.243
Rzeszów 2010	**0.781**	**0.682**	**0.750**	−0.299	−0.301	–
Rzeszów 2011	**0.591**	**0.490**	**0.354**	NS	NS	–
Rzeszów 2012	**0.608**	**0.352**	NS	NS	NS	–

NS—not significant; highlighted bold—positive correlation

### Synchronization

The synchronization of the patterns of spore seasons was high, as confirmed by statistically significant Spearman’s rank correlation coefficients (Table 4). The highest similarities of spore fluctuations were observed in Lublin where the values of the coefficients (*r*) ranged from 0.701 (between 2010 and 2012) to 0.810 (between 2011 and 2012). In Rzeszow, the synchronizations were the lowest, but nevertheless significant. The coefficients ranged from 0.465 to 0.620. The values of correlation coefficients obtained for Poznan were similar to those obtained for Rzeszow. There was a high synchronization of data obtained for different sites within the same season. The highest Spearman’s rank correlation coefficient (*r* = 0.821) was obtained for Lublin and Rzeszow in 2010, Lublin and Poznan in 2010, as well as for Lublin and Poznan in 2011 (*r* = 0.819) (Table 4).

**Table 4:** Spearman’s rank correlation coefficients between the patterns of *Cladosporium* spore seasons (*p* ≤ 0.05)

Monitoring site	Year	Lublin			Poznan			Rzeszow		
		2010	2011	2012	2010	2011	2012	2010	2011	2012
Lublin	2010		0.720	0.701	0.821	0.657	0.628	0.821	0.650	0.578
	2011	0.720		0.810	0.680	0.819	0.605	0.725	0.653	0.623
	2012	0.701	0.810		0.692	0.710	0.658	0.674	0.610	0.661
Poznan	2010	0.821	0.680	0.692		0.607	0.558	0.712	0.592	0.522
	2011	0.657	0.819	0.710	0.607		0.586	0.643	0.643	0.579
	2012	0.628	0.605	0.658	0.558	0.586		0.599	0.454	0.561
Rzeszow	2010	0.821	0.725	0.674	0.712	0.643	0.599		0.620	0.594
	2011	0.650	0.653	0.610	0.592	0.643	0.454	0.620		0.465
	2012	0.578	0.623	0.661	0.522	0.579	0.561	0.594	0.465	

Multidimensional cluster analysis, using Ward’s hierarchical clustering method, enabled the identification of monitoring sites with similar daily concentrations and the fluctuations of *Cladosporium* spores in the air. The dendrogram indicated four groups: the biggest similarity was observed among Rzeszow 2010 and 2011 and Poznan 2011, and then for Lublin 2011 and 2012 (Fig. 5). High degree of similarity was also obtained for the spore seasons of Rzeszow and Poznan 2012 as well as for Poznan and Lublin 2010, which were grouped in pairs, that were significantly different from the other site-years.

### Dynamics of spore seasons

**Fig. 6:**
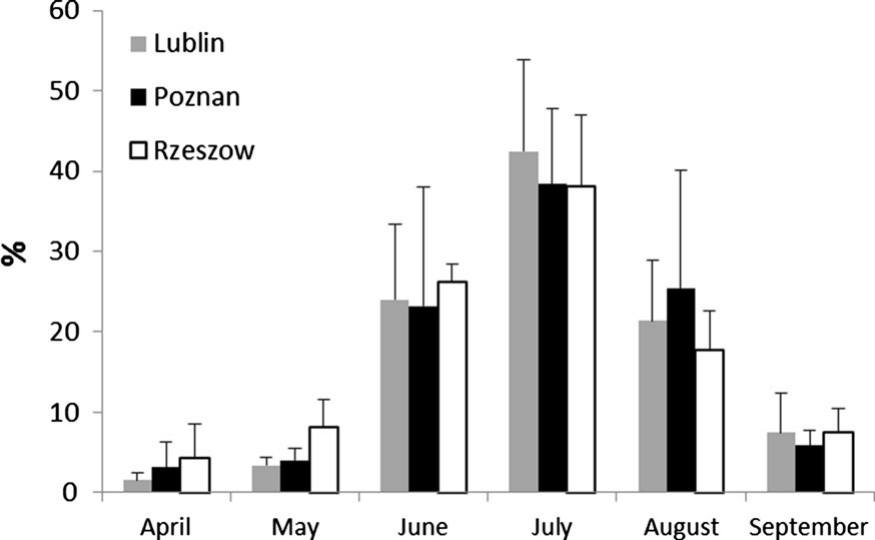
Mean percentages (±SD) of conidiospores of *Cladosporium* spp. in air (s m^−3^) of three Polish cities in 2010–2012

The mean percentages of total spore numbers per month were similar in all cities (Fig. 6). The lowest percentages of spores, below 5 %, were found in April. In May and September, the amounts of *Cladosporium* spores did not exceed 10 % and ranged from 3.4 % in Lublin to 8.2 % in Rzeszow, and both of these situations were found in May. In June, the amounts were nearly identical: 24.0, 23.2 and 26.2 % in Lublin, Poznan and Rzeszow, respectively. Comparable results were observed in August. In both months, Lublin and Poznan showed higher values of standard deviation of this parameter. The highest mean percentage of *Cladosporium* spores was found in July in all cities. These values were 42.4, 38.4 and 38.1 %, for Lublin, Poznan and Rzeszow, respectively (Fig. 6).

**Fig. 7:**
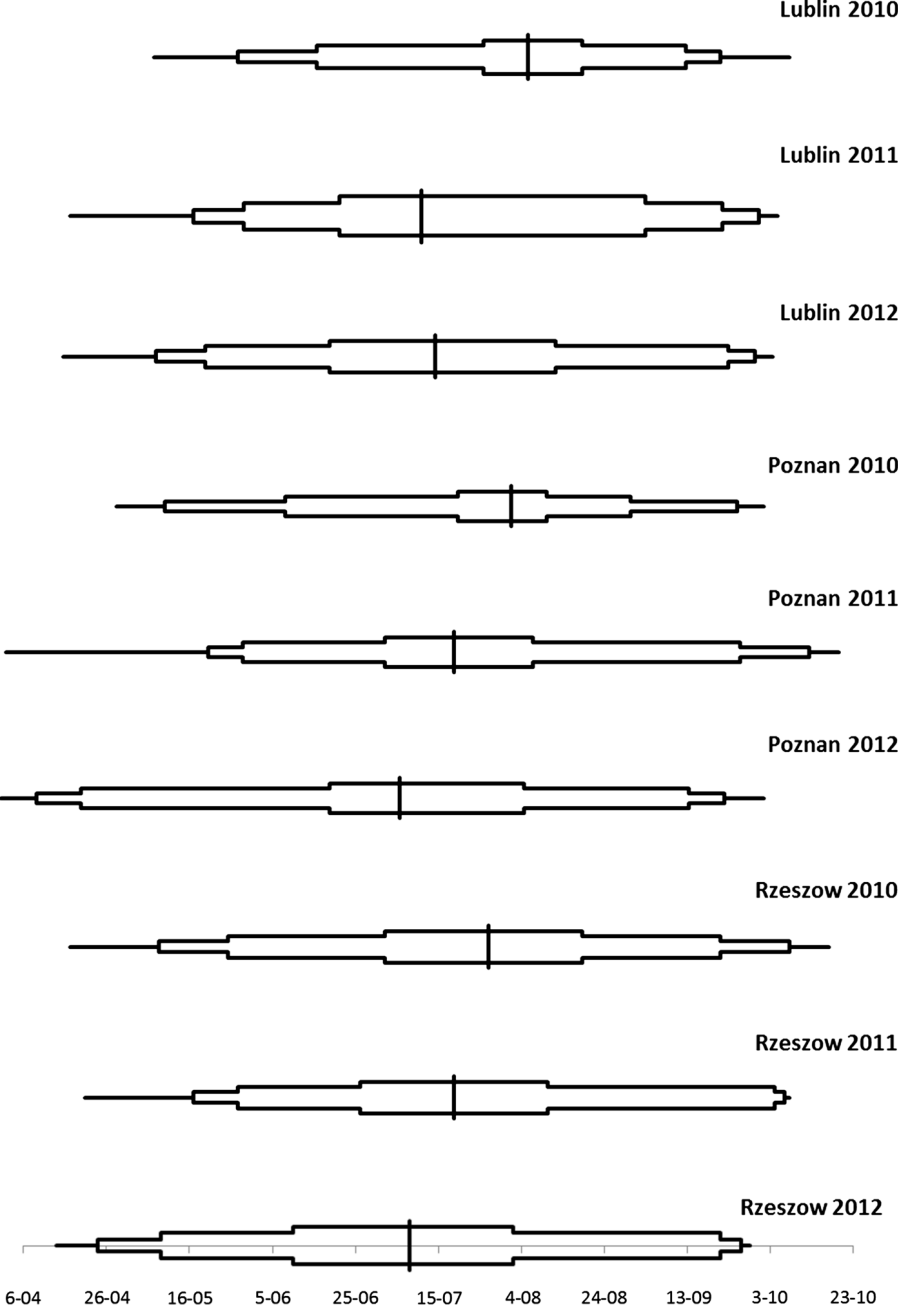
Differences between spore seasons of *Cladosporium* spp. in three cities over 3 years (2010–2012). Horizontal lines and boxes indicate eight stages of spore seasons: 1; 2.5; 5, 25; 75; 95; 97.5; and 99 %. The vertical line indicates the cumulative amount of 50 % of the spores

In Lublin and Poznan, the *Cladosporium* spore seasons showed significant differences between the years of the study (Fig. 7). Consecutive stages that represented the subsequent cumulative percentages of spores in the air per season, in most cases, differed in length. Exceptionally, in Lublin 2012 and in Poznan 2011 stages representing the cumulative percentages of spores from 25 to 95 % had similar duration. This phenomenon refers to the period of the highest spore concentrations and their constant presence in the air. The large similarity of spore seasons was observed in Lublin and Poznan in 2010. The spores within the range of 25–95 % were captured in a relatively short period of time, indicating that their release was very rapid (20–25 days). In many, but not in all cases, two halves of the season were almost symmetrical (Fig. 7).

Time of the occurrence of subsequent phases (Table 5) and the length of *Cladosporium* spore seasons (Table 6) were substantially different in the years of this study. In most cases, the cumulative phases of the first half of the season were observed earlier or even much earlier in subsequent years. In Lublin, all phases up to 50 % were noted earlier, from 3 to 24 days (Table 5). In Poznan, the phases 1 and 2.5 % were slightly delayed in time (from 1 to 5 days), but subsequently, all further phases until the accumulation of 75 % of spores were earlier, from 1 to 46 days. In Rzeszow, such phenomenon was not observed in 2011, but was clearly found in 2012. In contrast, the second half of the *Cladosporium* spore season was usually delayed. This phenomenon was the strongest in Lublin, where the delay ranged from 1 up to 26 days, depending on the year and phase of the spore season (Table 5).

**Table 5:** Differences in the dates of reaching the subsequent cumulative phases of *Cladosporium* spore seasons

Site/year difference	Cumulative phases of *Cladosporium* spore season									
	1	2.5	5	25	50	75	90	95	97.5	99
*Lublin*										
2010	7 May	24 May	9 June	13 July	22 July	2 Aug	14 Aug	23 Aug	30 Aug	13 Sept
2011–2010^a^	−11	−3	−8	−21	−12	−5	+1	+22	+23	+13
2012–2010^b^	−17	−13	−18	−24	−9	+7	+26	+25	+24	+14
2012–2011^c^	−6	−10	−10	−3	−3	−12	−17	+3	+1	+1
*Poznan*										
2010	2 May	13 May	9 June	18 July	30 July	7 Aug	15 Aug	26 Aug	19 Sept	25 Sept
2011–2010^a^	+5	+5	−15	−26	−23	−16	−1	−23	−2	−2
2012–2010^b^	+4	+1	−46	−23	−25	−5	+10	−13	−3	0
2012–2011^c^	−1	−4	−31	−3	−2	−10	+11	+5	−1	+2
*Rzeszow*										
2010	20 April	8 May	22 May	23 June	14 July	2 Aug	21 Aug	30 Aug	13 Sept	21 Sept
2011–2010^a^	+3	+7	+2	−5	−7	−7	+5	+11	−1	−8
2012–2010^b^	−6	−14	−13	−13	−6	0	+11	+22	+13	+7
2012–2011^c^	−9	−21	−15	−8	+1	−7	+6	+11	+14	−15

Positive number—the date of reaching the cumulative phase was later in the following year

Negative number—the date of reaching the cumulative phase was earlier in the following year (=acceleration)

^a^Difference of the date of reaching the cumulative phase, between 2011 and 2010 (no. of days)

^b^Difference of the date of reaching the cumulative phase, between 2012 and 2010 (no. of days)

^c^Difference of the date of reaching the cumulative phase, between 2012 and 2011 (no. of days)

**Table 6:** Differences in the length of the subsequent cumulative phases of *Cladosporium* spore seasons in experiment year and between years

	Length of phases in *Cladosporium* spore season					
	Year			Difference between years (no. of days)		
	2010	2011	2012	2011–2010^a^	2012–2010^b^	2012–2011^c^
*Lublin*						
1–99 %	129	153	160	+24	+31	+7
2.5–97.5 %	98	124	135	+26	+37	+11
5–95 %	54	105	118	+51	+64	+13
25–75 %	20	36	51	+16	+31	+15
*Poznan*						
1–99 %	146	169	172	+23	+26	+3
2.5–97.5 %	129	122	125	−7	−2	+3
5–95 %	78	101	137	+23	+59	+36
25–75 %	20	31	43	+11	+23	+12
*Rzeszow*						
1–99 %	154	143	167	−11	+13	+24
2.5–97.5 %	128	140	155	+12	+27	+15
5–95 %	59	69	83	+19	+24	+14
25–75 %	40	38	53	−2	+13	+15

Positive number—the cumulative phase was longer in the following year (=elongation)

Negative number—the cumulative phase was shorter in the following year

^a^Difference between 2011 and 2010 (no. of days)

^b^Difference between 2012 and 2010 (no. of days)

^c^Difference between 2012 and 2011 (no. of days)

An earlier start coupled with a later end caused the extension of *Cladosporium* spore seasons (Table 6). This clear trend was seen for both the core of the season (25–75 % of spores) and periods including the most season duration (5–95, 2.5–97.5 and 1–99 % of spores). The longest extension was observed in Lublin: the time from 5 % to 95 % of spore counts lasted 54 days in 2010, and it was 118 days in 2012 (64 days longer). Similarly, the difference between the same phases in the same years in Poznan was also substantial (59 days). The shortest value of this parameter was obtained in Rzeszow (24 days), even though a similar trend was found (Table 6).

## Discussion

Thermal conditions belong to the factors having the strongest influence on the spore concentration. The study of *Cladosporium* spore seasons in Poland was performed in a relatively short period of time (3 years), but it coincided with a period of significant warming of air masses. The mean raise of yearly air temperature was ranging from 0.9 °C in Lublin and Rzeszow, both located in east Poland, to 2.0 °C in Poznan, situated in west of the country. In east of Poland, it was comparable to the average global air temperature rise in the period of 1880–2012, reported by the Intergovernmental Panel on Climate Change, which accounted for 0.85 °C (IPCC 2013). In Poznan, a double of this value was achieved in the study period. It emboldened us to describe the trends in *Cladosporium* spore seasons observed in this period. We have found that under such conditions both mean and median values of total *Cladosporium* spore numbers significantly increased and the spore seasons were greatly accelerated and elongated.

The genus *Cladosporium* is composed of nearly 800 species (Dugan et al. 2004), varying in their life cycles, media preferences, abundance and timing of sporulation. The production of spores is species specific; as an example the experiments done by Harvey (1970) have shown that *C. herbarum*, *C. cladosporioides* and *C. shaerospermum* produced significantly more spores than *C. datum*, *C. resinae* and *C. macrocarpum*. The growth and sporulation of fungal cultures were decreased by wet conditions. Damialis et al. (2015a) registered higher spore production of *C. oxysporum* compared to *C. clarosporioides*, on average 3.5 × 10^6^ and 1.7 × 10^6^ per cm^3^ of agar medium. Such voluminous production of spores is then reflected in the enormous number of spores in the air. In our study, at each site-year, besides one exception (Rzeszow 2010) the daily concentration of *Cladosporium* exceeded 2000 spores per cubic metre on more than half days of spore monitoring.

The abundance of sporulation is associated with nutrient availability. In experimental conditions, *C. cladosporioides* doubled its spore production, when it was cultured on nutrient-rich agar (Damialis et al. 2015a). This explains why more spores of *Cladosporium* were repeatedly found in Poznan and Lublin, which are surrounded by multihectare fields of agricultural crops, serving as a source of media for the development of the studied fungi. A similar warming up of the weather in Lublin and Rzeszow resulted in a comparable increase in spore concentrations in the air; however, the mean spore numbers and their maximal concentrations were always higher in Lublin than in Rzeszow. The latter city is located in more wooded area, which does not supply *Cladosporium* with as many nutrients, as compared to areas of intensive agricultural fields. Similar tendencies supporting this hypothesis were previously observed in the same region for fungi of the genus *Alternaria* (Kasprzyk et al. 2013).

Abundant spore production and widespread occurrence of *Cladosporium* fungi resulted in a numerous presence of its spores in most of the areas, regardless of climate, altitude, vegetation and land use. In the entire study period, *Cladosporium* occurred in the air every day in all studied cities, except 1 day in Rzeszow in 2011 and 2012. Over 90 % days, the mean spore concentration exceeded 100 spores per cubic metre. Similar results were obtained by two separate aerobiological studies conducted in different regions of Iberian Peninsula (Rodriguez-Rajo et al. 2005; Aira et al. 2012). The monitoring of *Cladosporium* performed in five cities (Santiago, Amares, Qurense, Vigo and Trives) showed the spores were in the air almost every day. In many regions of Poland, *Cladosporium* constituted 83.8–90 % of all fungal material present in air samples (Stepalska et al. 1999; Kasprzyk and Worek 2006). Similarly, out of 23 fungal species found in Argentina, 85.7 % of spores belonged to the type *Cladosporium* (Negrin et al. 2007). It dominated also in the air of Thessaloniki, where the mean amount of spores reached 72 % (Damialis et al. 2015b). In Caxias do Sul, Brazil, located in a tropical pluvial bioclimate, the spores of *Cladosporium* dominated (32–34 %), but their percentage was clearly lower than in Europe (De Antoni Zoppas et al. 2006). Comparable results were obtained in Melbourne, Australia, where Mitakakis (2001) found 42 % of *Cladosporium* in aeroplankton. Aerobiological monitoring done in Spain distinguished two types of *Cladosporium* species (*C. herbarum* and *C. clarosporioides*), but together they constituted over 95 % of yearly sums of all detected spores (Rodriguez-Rajo et al. 2005). In the 1960s in Cardiff, UK, *C. herbarum* represented up to 70 % of total airborne *Cladosporium* spores (Harvey 1970).

Information that weather affects growth of mycelium, sporulation, transport and deposition of fungal spores was previously reported by the other authors (Stepalska and Wolek 2005; Grinn-Gofron and Strzelczak 2013). According to Harvey (1970), dry conditions favour sporulation and may double the number of *Cladosporium* spores. This relationship is also clearly visible in this study. Warming of air coincided with much lower precipitation, by 239 and 256 mm, respectively, in Poznan and Lublin, and as much as 422 mm in Rzeszow. Thus, changes of spore seasons were a combined effect of both warmer air temperature and lower rainfall. The decrease in wetness in Lublin and Poznan was mainly in spring (April–May), late summer and early autumn (August–September), but in Rzeszow it was observed over the whole study.

To some degree, the pattern of spore season reflects the type of climate. During this study, the patterns of spore seasons were typical for the warm temperate climate. In such conditions, the number of *Cladosporium* spores in the air gradually increases since mid-April in parallel to temperature, and it remains high or relatively high until the end of September (Konopinska 2004; Stepalska and Wolek 2005; Kasprzyk and Worek 2006). In Poland, the highest concentrations were usually noted in June–August, when the temperatures were high and rainfall was sufficient for fungal growth and sporulation. This study showed the same trend, but it was substantially magnified by warm and dry weather conditions. The monitoring of *Cladosporium* spores performed in the northern and eastern parts of the Iberian Peninsula, showed much earlier beginning of the season (February–March) and the spores occurrence in the air till the end of the calendar year (Aira et al. 2012). In southern Spain, the spore concentrations in the spring (April–June) and in early fall (September–October) were twice as high as in the summer. In July and August, the number of airborne spores decreased because of the complex of unfavourable environmental conditions. The temperature and insolation were very high, and the precipitation was extremely low, with long drought periods. In this case, the vegetation was greatly retarded, which affected fungal growth and sporulation due to the lack of many nutrients originating from host plants (Rodriguez-Rajo et al. 2005). According to Papkour et al. (2015), precipitation in proceeding periods may greatly affect fungal sporulation, and rainfall during 2 months prior to the observed period negatively affected spore concentration in two North American cities. It is therefore highly probable that high rainfall observed in Poznan in June and July 2011 and 2012 restricted further development and sporulation of *Cladosporium*; hence, the overall spore numbers in these 2 years were lower than expected.

The spore seasons started earlier and ended later at all monitoring sites of this study. The length of seasons and period of the highest spore concentrations were prolonged. It was evident both between 2010 and 2011 as well as between 2010 and 2012. The acceleration of the date of the maximum of spore count per cubic metre in subsequent years was 9 and 17 days in Lublin, 29 and 14 days in Poznan, 16 and 40 days in Rzeszow, respectively, in 2011 and 2012. All in all, the acceleration of the date of the maximum *Cladosporium* spore concentration per cubic metre of the air in Lublin, Poznan and Rzeszow was 26, 43 and 56 days, which gives an average of 42 days. The earliest start of the spore season in Rzeszow, observed on 21 May 2012, was at first glance quite unexpected, as the city is located in the east of Poland. However, the analysis of weather parameters showed that although the mean air temperature in 2012 was by 0.5 °C higher in Poznan (west Poland) than in Rzeszow, the start of the spore season (April) was warmer in Rzeszow by more than 1 °C, which was directly preceding the abundant production and release of *Cladosporium* spores in May 2012. It is very likely that the earlier start of the spore season resulted from the earlier start of the vegetation of plants, which are the source of nutrients to fungi. At the discussed period (spring 2012), the rainfall in Rzeszow was 70 mm higher than in Poznan, which surely pushed the plant vegetation forward.

The investigation of spring phenology of basidiomycetes indicated earlier start of fruiting, linked with a global increase in temperature (Kauserud et al. 2010). The most important were temperatures of previous summer and winter. Damialis et al. (2015b) also pointed temperature as the most important factor influencing change in the seasons of airborne spores. In Greece, during 15 years of monitoring the annual airborne *Cladosporium* spore concentrations slightly decreased, while mean air temperature increased. However, in contrast to several fungal taxa, the weak tendency of earlier onset of spore season and longer duration were noted for *Cladosporium*. Long-term aeromycological monitoring conducted in the other two cities of Poland (Krakow and Szczecin) showed an earlier start of the season in Krakow and the decrease in annual spore sum in Szczecin (Grinn-Gofron et al. 2015). Laboratory data obtained recently by Damialis et al. (2015a) greatly explain the results obtained in our study. In contrast to numerous fungal taxa, the authors showed the unchanged production of the spores of *C.cladosporioides*. In the light of our recent data, based on precise molecular analyses (Kaczmarek et al., unpublished) this *Cladosporium* species is very abundant in the air over Poznan and Rzeszow. The raise of the number of conidia, the acceleration and elongation of *Cladosporium* spore seasons will surely have an adverse effect on human health. It may also lead to lowering of the yield of plants attacked by the pathogens of this fungal genus (such as *C. fulvum*) or decrease the quality of seeds and other plant organs inhabited by these necrotrophs. It is therefore very important to be aware of the impending danger and of the possible bad scenarios.
